# Bilateral Ulnar Neuropathy Secondary to Chronic Pentazocine Injections: A Rare Neurological Sequela of Substance Abuse

**DOI:** 10.1002/ccr3.73124

**Published:** 2026-07-19

**Authors:** Malaika Khan, Samra Aleem, Muhammad Taha, Malik W. Z. Khan, Sajjad Ullah, Kamil Ahmad Kamil

**Affiliations:** ^1^ Khyber Medical College Peshawar KPK Pakistan; ^2^ Yale University School of Medicine New Haven Connecticut USA; ^3^ Department of Neurosurgery Khyber Teaching Hospital Peshawar Pakistan; ^4^ Nasrat Bashir Curative Hospital Kandahar Afghanistan

**Keywords:** case report, drug addiction, substance abuse, ulnar nerve entrapment, ulnar neuropathy

## Abstract

Ulnar neuropathy (UN) is the second most common entrapment neuropathy, typically resulting from trauma, fractures, compression, dislocations, repetitive elbow movements, or congenital malformations secondary to previous injuries. However, it may also arise as a complication of substance abuse. We report the case of a 45‐year‐old male with bilateral ulnar neuropathy secondary to 22 years of intravenous pentazocine (Sosegon) abuse, highlighting repeated local tissue injury and fibrosis as potential pathogenic factors. The patient presented with progressive swelling, numbness, and pain in both upper extremities, along with a bilateral flexion contracture of the left fourth and fifth digits. Nerve conduction studies (NCS) revealed severe left ulnar neuropathy at the elbow distal to the branch of the flexor carpi ulnaris, and moderate‐to‐severe right ulnar neuropathy at the elbow proximal to the same branch. Needle electromyography (EMG) demonstrated significant denervation in muscles innervated by the ulnar nerve. After preoperative evaluation, the patient underwent successful ulnar nerve transposition followed by physiotherapy, resulting in symptomatic improvement at six‐month follow‐up. This case highlights chronic pentazocine abuse as a rare but significant cause of bilateral ulnar neuropathy, likely due to repeated local trauma and fibrosis. Early recognition and a multidisciplinary management strategy involving neurosurgery, addiction medicine, and physiotherapy to address both the neurological sequelae and the underlying substance abuse are essential to optimizing long‐term functional and clinical outcomes.

## Key Clinical Message (KCM)/Learning Points

- Bilateral ulnar neuropathy is rare and may indicate systemic or external causes like substance abuse.
- Intravenous drug abuse can cause it via nerve trauma, compression, or toxicity.
- Early diagnosis and, if needed, surgical intervention such as ulnar nerve transposition are vital to prevent permanent damage and restore function.

## Graphical abstract

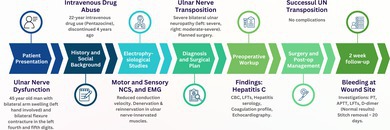

## 1 Introduction

Ulnar neuropathy at the elbow, with a prevalence of 0.6%–0.8% and an incidence of 20–25 per 100,000 person‐years, is the second most common entrapment neuropathy after carpal tunnel syndrome (CTS) [1]. One of the primary causes of ulnar neuropathy is compression of the ulnar nerve at any point along its course. This compression can result from various anatomical variations and pathological factors such as accessory muscles or ligamentous structures, repetitive mechanical stress, trauma, inflammatory conditions, or systemic conditions such as diabetes mellitus [2, 3, 4, 5, 6, 7, 8]. Some studies suggest a higher prevalence of ulnar neuropathy in the left upper limb, possibly due to frequent use for body stabilization, including leaning and extension, thereby subjecting the ulnar nerve to sustained pressure. In contrast, the right hand is generally more involved in fine motor activities, which may explain its lower susceptibility, as noted in previous research [9].

Pentazocine was developed as an effective analgesic with partial agonist and antagonist activity at the μ‐opioid receptors, thus possessing addictive potential [10]. Opioids like heroin have been shown to impair the balance between oxidation and anti‐oxidation and disrupt the Bcl‐2/Bax ratio, affecting cell survival and neuronal death [11, 12]. Studies have highlighted the long‐term effects of heroin abuse, causing activation of microglial cells and therefore leading to inflammation and neurotoxicity [13].

Existing literature on bilateral ulnar neuropathy has reported various conditions as risk factors, including tardive dyskinesia in a patient receiving high‐dose oral anti‐psychotic [14], prolonged engagement in computer‐based tasks [15], or a complication of Charcot arthropathy [16]. Although drug‐induced peripheral neuropathies are increasingly recognized, their role in entrapment neuropathies remains underreported. Pentazocine abuse is known to cause severe local tissue damage, fibrosis, and ischemia; however, its association with bilateral ulnar neuropathy is not adequately documented.

This case fills an important knowledge gap by highlighting a rare and overlooked cause of ulnar neuropathy and emphasizes the severe neurological complications and risks associated with chronic intravenous drug abuse, particularly the repeated needle trauma that can lead to significant nerve injury and functional impairment.

## 2 Case Description

A 45‐year‐old male laboratory technician presented to the Neurosurgery Outpatient Department with complaints of bilateral upper limb swelling, more pronounced in the left hand. The swelling was firm, non‐compressible, and reportedly worsened with sun exposure. Notably, the patient did not report pain in the affected areas. Clinical examination revealed bilateral flexion contractures of the left fourth and fifth digits, consistent with ulnar nerve involvement. Upon further history‐taking, the patient disclosed a six‐year history of bilateral hand numbness and an 8–10‐year history of flexion contractures. His social history was significant for 22 years of intravenous (IV) pentazocine abuse, which he had discontinued four years prior. Each injection contained 30 mg of the drug. Initially, he used 4–5 injections per day, gradually escalating over the years to 20–25 injections per day. Injections were frequently administered at bilateral upper limb sites, particularly around the elbows and forearms, often at repeated venipuncture sites. Pentazocine, a narcotic analgesic, is commonly used as an adjunct to general anesthesia but carries a high potential for addiction.

The patient had no history of diabetes mellitus, hypertension, joint pain, rheumatoid arthritis, fractures, or any significant trauma that could explain the neuropathy. Additionally, no soft tissue masses or palpable abnormalities were detected at the elbow upon clinical examination. His past surgical history included a tonsillectomy and appendectomy, performed 15 years ago. Family history was notable for diabetes mellitus in his mother and prostate cancer in his father. A significant aspect of the patient's history was multiple hospitalizations due to injection‐site complications. The patient provided an NCS and EMG report conducted three years prior, which showed:

- Motor NCS: Reduced conduction velocity in the right ulnar nerve below or around the elbow, suggesting demyelination. No measurable responses were obtained for the left ulnar nerve, indicating severe neuropathy (Table 1).
- Sensory NCS: Sensory nerve testing showed no recordable signals (SNAPS), indicating severe sensory nerve damage in both limbs (Table 2).
- Needle EMG: Showed loss of nerve supply (denervation) with signs of partial recovery (re‐innervation) in muscles supplied by the ulnar nerve on both sides.

**TABLE 1:** Motor nerve conduction study (NCS) report—Summary of motor conduction parameters for the ulnar nerve, including latency, amplitude, conduction velocity, and segmental differences.

Site	Latency	Amplitude	Segment	Latency Difference	Distance	Conduction Velocity	Normal Values
*Ulnar Nerve (Right)*							
Wrist	3.7 ms	1.1 mV	ADM—Wrist	3.7 ms	mm	m/s	Latency ≤ 3.5 ms; Amplitude ≥ 6 mV
Below elbow	7.0 ms	0.8 mV	Wrist‐ Below elbow	3.3 ms	240 mm	73 m/s	CV ≥ 50 m/s
Above elbow	9.4 ms	0.6 mV	Below elbow‐ Above elbow	2.4 ms	100 mm	42 m/s	CV ≥ 50 m/s
*Ulnar Nerve (Left)*							
Wrist	NR ms	NR mV	ADM—Wrist	—	—	m/s	Latency ≤ 3.5 ms; Amplitude ≥ 6 mV
Below elbow	NR ms	NR mV	Wrist‐ Below elbow	—	—	m/s	CV ≥ 50 m/s
Above elbow	NR ms	NR mV	Below elbow‐ Above elbow	—	—	m/s	CV ≥ 50 m/s

*Note:* The marked reduction in conduction velocity and amplitude in the right ulnar nerve, along with absent responses in the left ulnar nerve, is consistent with severe bilateral ulnar neuropathy, with more pronounced involvement on the left side. Normal reference values are provided for comparison.

Abbreviations: ADM, Abductor digiti minimi; CV, Conduction velocity; m/s, Meter per second; mm, Millimeter; ms, Millisecond; mv, Millivolt; NR, No response.

**TABLE 2:** Sensory nerve conduction study (NCS) report—Sensory conduction findings of the ulnar nerve.

Side	Site	Onset Latency (ms)	Peak Latency (ms)	Amplitude (μV)	Conduction Velocity	Reference Value
Right	Wrist (Ulnar)	NR	NR	NR	NR	Onset ≤ 3.5 ms; Amplitude ≥ 10 μV
Left	Wrist (Ulnar)	NR	NR	NR	NR	Onset ≤ 3.5 ms; Amplitude ≥ 10 μV

*Note:* The lack of recordable sensory nerve action potentials (SNAPs) in both limbs suggests severe bilateral sensory involvement, supporting the diagnosis of advanced ulnar neuropathy. Normal reference values are provided for comparison.

Abbreviations: mm, Millimeter; Ms, Millisecond; NR, No response; μV, Microvolt.

On day six, the patient underwent preoperative laboratory investigations, including complete blood count (CBC), hepatitis serology, liver function tests (LFTs), electrolyte levels, coagulation profile, and echocardiography. The results revealed positive Hepatitis C serology, mildly elevated ALT/SGPT and alkaline phosphatase, and no other significant abnormalities.

**FIGURE 1:**
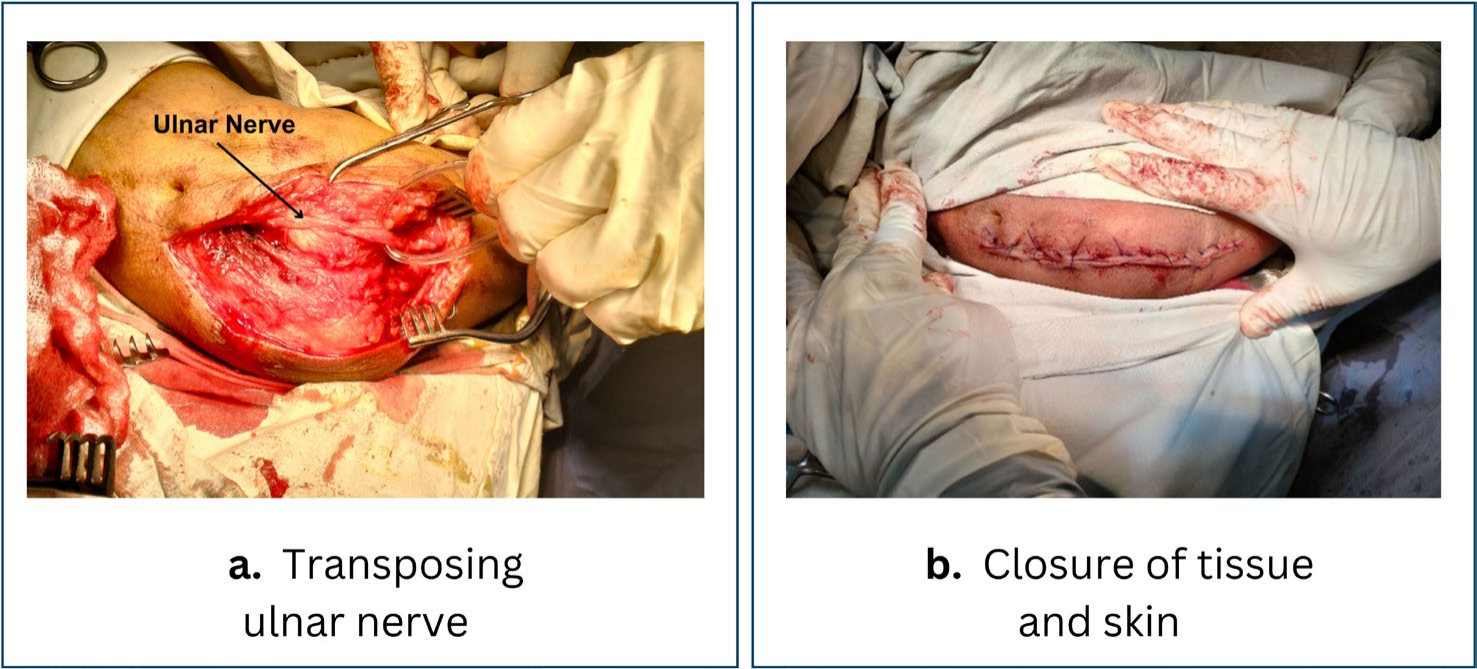
Ulnar Nerve Transposition Surgery—Schematic representation of the surgical procedure in which the ulnar nerve is relocated from behind the elbow to the front to relieve compression.

The patient underwent ulnar nerve transposition on day nine. This procedure involves relocating the ulnar nerve from behind the elbow to a safer position on the front to relieve compression during movement. The surgery was successfully performed without complications. Figure 1 shows the schematic representation of the surgical procedure. Postoperatively, the patient received intravenous fluids, analgesics, antibiotics (cefoperazone‐sulbactam), and gastro‐protective therapy (omeprazole). On day 10, he was discharged on oral antibiotics, anti‐inflammatory medications, and neurotropic supplementation (mecobalamin).

At the two‐week follow‐up, suture removal was delayed due to mild bleeding from the surgical site. Coagulation (PT, APTT, and D‐dimer) and liver function tests were performed and were within normal limits. The patient was discharged on Tranexamic acid to control bleeding. Sutures were successfully removed after 20 days. Physiotherapy was recommended to improve mobility. The patient did not experience significant postoperative complications.

**FIGURE 2:**
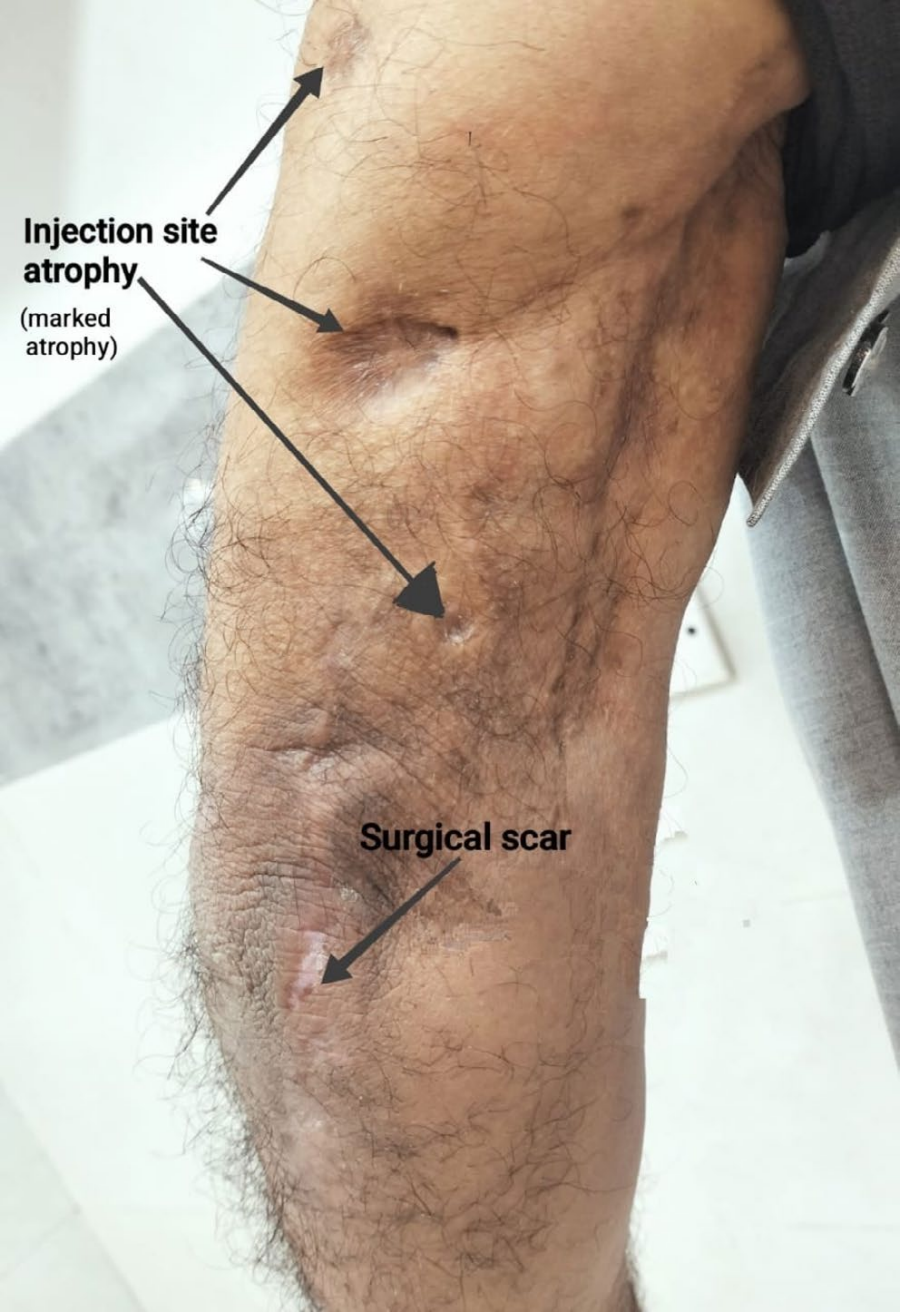
Postoperative Condition of the Patient's Arm—Clinical image depicting the postoperative state of the affected limb, showing a well‐healed surgical site. Marked atrophy is visible at previous injection sites due to vascular compromise from repeated trauma.

**FIGURE 3:**
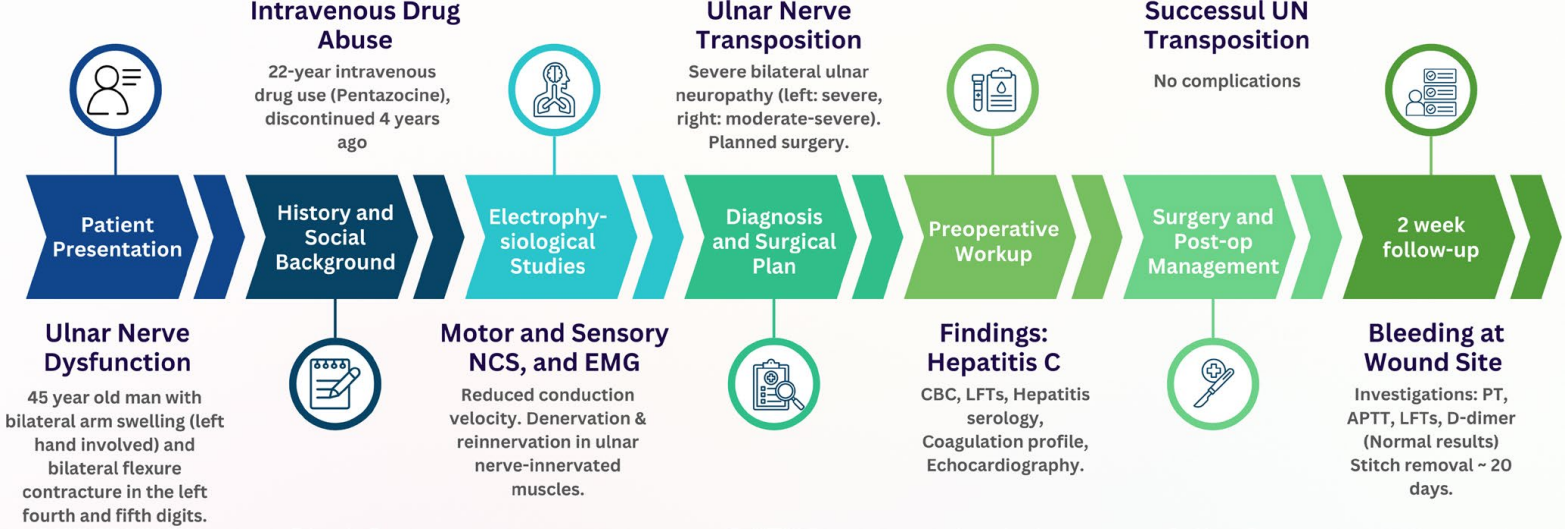
Bilateral Ulnar Neuropathy Management Workflow—A visual representation summarizing the patient's diagnostic workup, treatment course, surgical intervention, and follow‐up progression.

Figures 2 and 3 illustrate the postoperative condition and the overall management workflow from diagnosis to recovery.

Table 3 illustrates the timeline table about the chronological progression of clinical events and management.

**TABLE 3:** Timeline table about chronological progression of clinical events and management (years of abuse, onset of symptoms, interventions, follow‐up).

TIME FRAME	EVENT
22 Years Prior	Initiation Of IV Pentazocine Abuse
8–10 Years Prior	Development Of Bilateral Hand Flexion Contractures
6 Years Prior	Onset Of Bilateral Hand Numbness
4 Years Prior	Abrupt Cessation Of Pentazocine Abuse
3 Years Prior	NCS/EMG Showing Severe Bilateral Ulnar Neuropathy
Day 0	Presentation To Neurosurgery OPD
Day 6	Preoperative Labs: Hepatitis C Detected
Day 9	Ulnar Nerve Transposition Surgery
2 Weeks Post‐op	Delayed Stitch Removal Due To Bleeding
20 Days Post‐op	Successful Stitch Removal
6 Months Post‐op	Improvement In Numbness; Physiotherapy Advised

## 3 Discussion

Ulnar neuropathy at the elbow is the second most common entrapment neuropathy after CTS [9]. Due to its superficial anatomical location, the ulnar nerve is highly vulnerable to mechanical compression and injury. Common causes include prolonged elbow flexion, sustained pressures, prior trauma, structural abnormalities, other systemic conditions, including arthritis or, in some cases, idiopathic causes. Ulnar neuropathy is also frequently observed in patients with chronic stroke [17]. Older male patients, particularly those with underlying conditions such as diabetes, are at a greater risk of developing bilateral ulnar neuropathy compared to unilateral [9].

The diagnosis of ulnar neuropathy is based on clinical findings, including sensory and motor impairments in the ulnar nerve's distribution in the hand. This includes localized sensory disturbances in the medial hand, including the fourth and fifth digits, and weakness or atrophy of intrinsic hand muscles. EMG and ultrasonography serve as confirmatory diagnostic tools [18].

### 3.1 Comparison With Previous Bilateral Ulnar Neuropathy Cases

Previous reports have described bilateral ulnar neuropathy in conditions such as tardive dyskinesia [14], prolonged laptop and computer use [15], and anatomical compression such as Charcot arthropathy [16]. While some studies used quantitative grading systems such as McGowan classification or Medical Research Council (MRC) scoring to quantify the nerve injury and muscle weakness, our diagnosis of bilateral ulnar was primarily supported by the patient's medical history, physical examination, and relied primarily on the qualitative assessment, such as EMG and NCS.

The symptoms like hand numbness, flexion contractures, weakness, and sensory deficits caused by other drugs like heroin, and due to long‐term exposure to high doses of the antipsychotic drug Haloperidol have also been reported in previously published studies [14, 19]. Dekelver et al., in their case report, reported numbness and tingling in the fourth and fifth digits of both hands. The contrasting presentation of the patient in this case was that there was tenderness and pain just proximal to the medial epicondyle, whereas in our case, there was no tenderness or pain. This might be due to the fact that in our case, the ulnar neuropathy was due to IV drug abuse rather than direct compression, whereas in Deklver et al.'s case report, it was due to the compression of the ulnar nerve by Anconeus epitrochliaris [20].

Clinically, the presentation was comparable to prior reports, but there has been no prior bilateral ulnar neuropathy case documented that is a consequence of more than two decades of constant IV pentazocine abuse. The unique feature of the case discussed here was the chronicity of exposure, the absence of alternative systemic or metabolic causes, and the electrophysiological confirmation.

Recent literature has documented foreign body‐induced granulomatous inflammation and vascular damage following intravenous injection of crushed oral opioid formulations such as pentazocine, hydrocodone, and oxycodone. Their IV administration has been demonstrated to introduce insoluble excipients such as talc and microcrystalline cellulose into the bloodstream that may induce chronic inflammatory and fibrotic changes. Nagarajan et al. described pulmonary hypertension secondary to talc granulomatosis in a 25‐year‐old male with a 5‐year history of polysubstance abuse [21]. Similarly, other case reports and autopsy‐based studies have demonstrated foreign body granulomatosis reactions and vascular remodeling resulting from injected opioid excipients [22, 23]. This provided the pathological evidence of vascular granulomatosis and remodeling induced by the injection of opioid tablets.

While these reports mostly focus on pulmonary involvement, the fundamental processes of foreign body‐mediated inflammation, vascular damage, and fibrosis are pertinent to peripheral tissues as well. In the present case, similar localized inflammatory and fibrotic responses surrounding the neurovascular structures at the elbow may have been caused by frequent IV pentazocine abuse, which may have contributed to bilateral ulnar nerve compression and neuropathy. This interpretation is further supported by a recent review highlighting the fact that opioid exposure and opioid‐related complications might entail peripheral nervous system pathology, including structural and inflammatory processes of nerve damage, in addition to effects on the central nervous system [24].

### 3.2 Pathophysiological Link Between IV Pentazocine and Neuropathy

IV drug abuse has been associated with peripheral neuropathies through several mechanisms. In this case, the patient had a 22‐year history of IV pentazocine abuse, with 6 years of persistent bilateral hand numbness and firm, non‐compressible swelling.

Potential mechanisms include repeated direct perineural trauma from injections, prolonged immobility during intoxication leading to sustained compression neuropathy at the cubital tunnel (elbow) or Guyon's canal (wrist), and substance‐related neurotoxicity causing both metabolic and vascular damage to peripheral nerves [25].

The majority of the previously published studies reporting drug‐induced neuropathies involve drugs like isoniazid, haloperidol, and chemotherapeutics. The underlying mechanisms of these drugs include axonal degeneration, interference with neurotransmitter metabolism, or mitochondrial toxicity [25], whereas in pentazocine abuse, the pathology is mainly due to localized fibrosis from insoluble excipients, opioid‐associated axonal injury, vascular compromise, and recurrent perineural trauma.

**FIGURE 4:**
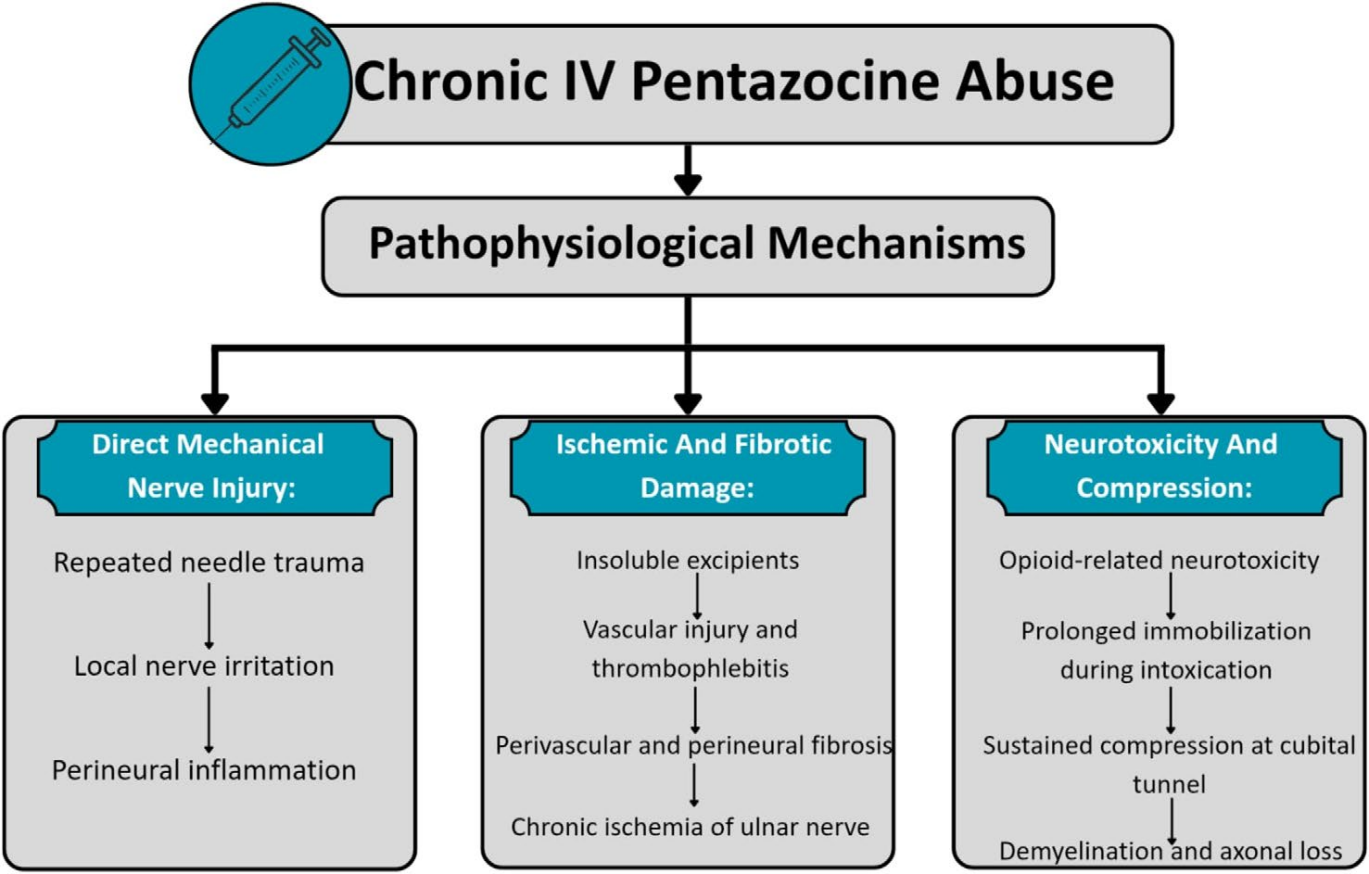
Proposed pathophysiological mechanisms of ulnar neuropathy associated with chronic IV pentazocine abuse—An illustration of proposed mechanisms of ulnar neuropathy caused by IV pentazocine abuse, including direct needle‐induced nerve injury, ischemic and fibrotic damage, and opioid‐related neurotoxicity and compression.

Figure 4 illustrates the pathophysiological mechanisms of ulnar neuropathy associated with chronic IV pentazocine abuse.

### 3.3 Surgical Outcomes and Rehabilitation Relevance

Management of ulnar neuropathy depends on the severity of symptoms. While conservative measures such as physical therapy, splinting, and nonsteroidal anti‐inflammatory drugs (NSAIDs) may be effective in mild cases to limit excessive movement and to alleviate pain and swelling, in cases where symptoms persist or progress, surgical intervention is considered.

Surgical management of ulnar neuropathy involves decompression procedures that may be performed in situ or with nerve transposition. In situ decompression involves releasing the nerve from compressive structures while keeping it in its original anatomical position. Conversely, decompression with transposition involves relocating the ulnar nerve to a less vulnerable position. In this patient, transposition was chosen to relieve compression and reposition the ulnar nerve anterior to the elbow, thereby reducing tension during flexion and minimizing recurrent mechanical stress [26, 27, 28, 29, 30, 31].

### 3.4 Clinical Implications for Substance‐Abuse Screening

The case discussed in this case report describes that bilateral ulnar neuropathy can result from the long‐term injection practices, and thus, pentazocine abuse should also be taken into account while performing the differential diagnosis of unexplained upper limb neuropathies because it can mimic the metabolic and systemic causes of bilateral entrapment neuropathies.

This case contributes to the existing body of knowledge by detailing a unique presentation of bilateral ulnar neuropathy secondary to IV pentazocine abuse, highlighting both the pathophysiological mechanisms and surgical treatment options.

## 4 Limitations

This case report has several limitations. The postoperative recovery was primarily described in clinical terms, such as contracture relief, reduced numbness, and improved hand mobility after physiotherapy. However, muscle functioning outcomes and standardized scoring systems like the Disabilities of the Arm, Shoulder, and Hand (DASH) score or Medical Research Council (MRC) muscle grading were not incorporated; they would have further strengthened the evidence.

Additionally, long‐term electrophysiological data and radiological correlation (ultrasound/MRI) were not performed to evaluate nerve morphology and decompression status. Future studies, such as case series or prospective studies with standardized clinical, electrophysiological, and imaging‐based outcome measures, would help better establish the dose‐injury relation and long‐term prognosis and management in similar patients.

## 5 Conclusions

This case highlights a rare presentation of bilateral ulnar neuropathy secondary to chronic IV pentazocine abuse. Repeated local injections likely contributed to perineural trauma, fibrosis, and progressive nerve compression. Ulnar nerve transposition proved to be an effective treatment, leading to significant symptomatic relief. Early recognition and a multidisciplinary management strategy, including neurosurgery, addiction medicine, and physiotherapy, are needed to address the underlying substance abuse, improving long‐term functional and clinical outcomes in IV drug–related ulnar neuropathy patients.

## Author Contributions

**Malaika Khan:** conceptualization, data curation, formal analysis, investigation, methodology, resources, visualization, writing – original draft, writing – review and editing. **Samra Aleem:** conceptualization, data curation, formal analysis, investigation, methodology, resources, visualization, writing – original draft, writing – review and editing. **Muhammad Taha:** data curation, investigation, methodology, visualization, writing – original draft, writing – review and editing. **Malik W. Z. Khan:** data curation, formal analysis, investigation, methodology, validation, visualization, writing – review and editing. **Sajjad Ullah:** conceptualization, data curation, project administration, resources, supervision, validation, visualization, writing – review and editing. **Kamil Ahmad Kamil:** project administration, writing – review and editing.

## Funding

The authors have nothing to report.

## Ethics Statement

This study was conducted in accordance with ethical guidelines, and consent for publication was obtained.

## Consent

Written informed consent was obtained from the patient for the publication of this case report and associated images, in compliance with the journal's consent policy. No personal information, such as name, address, or occupation, was disclosed.

## Conflicts of Interest

The authors declare no conflicts of interest.

## Data Availability

All relevant data are included in this article.
